# Convolutional Neural Networks in the Inspection of Serrasalmids (Characiformes) Fingerlings

**DOI:** 10.3390/ani14040606

**Published:** 2024-02-13

**Authors:** Marília Parreira Fernandes, Adriano Carvalho Costa, Heyde Francielle do Carmo França, Alene Santos Souza, Pedro Henrique de Oliveira Viadanna, Lessandro do Carmo Lima, Liege Dauny Horn, Matheus Barp Pierozan, Isabel Rodrigues de Rezende, Rafaella Machado dos S. de Medeiros, Bruno Moraes Braganholo, Lucas Oliveira Pereira da Silva, Jean Marc Nacife, Kátia Aparecida de Pinho Costa, Marco Antônio Pereira da Silva, Rodrigo Fortunato de Oliveira

**Affiliations:** 1 Federal Institute of Education, Science and Technology of Goiás (IF Goiano)—Campus Rio Verde, Goiana South Highway, Km 01, Rio Verde 75901-970, GO, Brazil; heyde.franca@ifgoiano.edu.br (H.F.d.C.F.); alenesantos47@gmail.com (A.S.S.); lessandro_lima@hotmail.com (L.d.C.L.); liegedouny@gmail.com (L.D.H.); mathebp@hotmail.com (M.B.P.); isabel.r.rezende@gmail.com (I.R.d.R.); rafaellamedeiros1@live.com (R.M.d.S.d.M.); brunombraganholo@gmail.com (B.M.B.); lucas.oliveira.freak@gmail.com (L.O.P.d.S.); jean.nacife@ifgoiano.edu.br (J.M.N.); katia.costa@ifgoiano.edu.br (K.A.d.P.C.); marco.antonio@ifgoiano.edu.br (M.A.P.d.S.); fortunatorodrigo@ymail.com (R.F.d.O.); 2 School of Biological Sciences, College of Arts and Sciences, Washington State University, Pullman, WA 99163, USA; pedroh1986@gmail.com

**Keywords:** aquaculture, Internet of Things, neural network

## Abstract

**Simple Summary:**

Artificial intelligence tools, such as convolutional neural networks, are used in tracking and counting animals, species identification, and measurement of morphometric data in order to optimize operations and minimize the animals’ stress and physical injuries when they are handled, which lead to an increase in disease and mortality in livestock. This study aims to evaluate and understand the effectiveness of counting different quantities of Serrasalmidae fingerlings by means of images using neural networks. These are promising tools to strengthen round fish farming, an important species for South American aquaculture, in order to increase production efficiency, profitability, and the transparency in the commercialization of fingerlings.

**Abstract:**

Aquaculture produces more than 122 million tons of fish globally. Among the several economically important species are the Serrasalmidae, which are valued for their nutritional and sensory characteristics. To meet the growing demand, there is a need for automation and accuracy of processes, at a lower cost. Convolutional neural networks (CNNs) are a viable alternative for automation, reducing human intervention, work time, errors, and production costs. Therefore, the objective of this work is to evaluate the efficacy of convolutional neural networks (CNNs) in counting round fish fingerlings (Serrasalmidae) at different densities using 390 color photographs in an illuminated environment. The photographs were submitted to two convolutional neural networks for object detection: one model was adapted from a pre-trained CNN and the other was an online platform based on AutoML. The metrics used for performance evaluation were precision (P), recall (R), accuracy (A), and F1-Score. In conclusion, convolutional neural networks (CNNs) are effective tools for detecting and counting fish. The pre-trained CNN demonstrated outstanding performance in identifying fish fingerlings, achieving accuracy, precision, and recall rates of 99% or higher, regardless of fish density. On the other hand, the AutoML exhibited reduced accuracy and recall rates as the number of fish increased.

## 1. Introduction

The production of aquatic organisms reached 122.6 million tons in 2020, a record for the aquaculture sector [1]. This growth is mainly due to increasing demand from the population, and in order to meet it, scientific research and technological innovations have been developed [2]. Among these, the most widespread and applied ones are those that generate market value, such as improvements in agility, accuracy, performance, cost reduction, convenience and ease of management, and traceability and/or process control through automation [3].

The counting of fish is essential for monitoring, control, and traceability. This is usually carried out by sampling using containers according to the species and average weight of the animals [4]. However, there is great variation in weight for most farmed species, even if size classification is performed, which results in errors in counts completed through sampling. This process has also been accomplished manually, which causes stress to the fish and laborers, it is time-consuming, and it is subject to human error [5]. To minimize such problems, there is a global trend toward the implementation of the Internet of Things (IoT) and artificial intelligence (AI) for automation and traceability in aquaculture, providing greater accuracy in the quantification of the flock, greater health control, and food safety for the consumer [6].

Artificial intelligence tools, such as connectionist techniques or artificial neural networks (ANNs), simulate biological synapses, which enables learning for recognition, counting, and classification of objects according to the organization and interaction of neurons [7,8]. The first ANNs presented limitations in processing large amounts of visual data. Therefore, analogously, convolutional neural networks (CNNs) emerged [7]. Focused on image analysis, CNNs have provided advances in fish identification, biometrics [9,10], and marine ecology [11,12]. In these neural networks, the classification, identification, and detection of features occur from sequential convolution layers, which allow for learning independence in each convolution. The extraction of the main features of the image without being fully connected in layers, provides a lightness in the connections in order to facilitate and improve the performance in recognizing objects in images [13,14,15].

In aquaculture, these technologies are observed in the tracking and counting of animals, species identification, and measurement of morphometric data. These technologies optimize operations and minimize the animals’ stress and physical injuries when they are handled, leading to a decrease in diseases and mortality in fish stock [6,16]. In the scientific literature, methods of counting and tracking fish by images obtained in aquatic environments are considered less invasive and more effective than manual or automated methods available on the market, with accuracy above 90% [17].

The number of objects contained in the image can affect this accuracy, especially in situations where the objects are overlapping and aggregated [18]. In fish fingerlings, the distribution of animals in the environment are not uniform, and this overlapping and aggregation of animals occur more frequently as the density of animals increases.

Studies evaluating the effectiveness of CNNs in counting different quantities of fry are still scarce in the scientific literature, especially for round fish (Serrasalmidae; Characiformes), despite the importance of these species for aquaculture in Latin America [19,20]. Among the Serrasalmidae species, those of economic importance are pacu, pirapitinga, tambaqui, and their respective hybrids due to the nutritional and sensory characteristics of their meat, which appeal to consumers [21,22]. In view of the above, the aim of this research is to evaluate the effectiveness of counting different quantities of Serrasalmidae fry by means of images using CNNs.

## 2. Materials and Methods

### 2.1. Database

**Figure 1:**
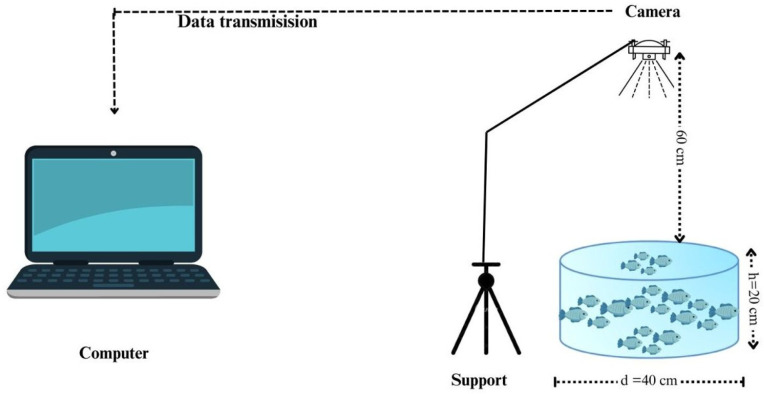
Fingerling image collection platform.

The dataset was obtained at the headquarters of the company “Alevinos Rio Verde”, located in the municipality of Rio Verde, state of Goiás, Brazil. The fingerlings, approximately three centimeters in length, were placed in blue-bottom 25 L containers with a 40 cm diameter and illuminated with LED light for sharpness, as shown in Figure 1.

**Figure 2:**
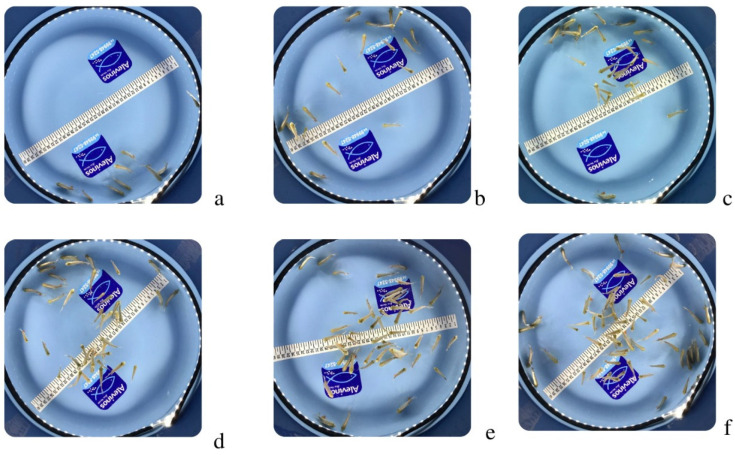
Images of the dataset with 9 (**a**), 20 (**b**), 30 (**c**), 40 (**d**), 50 (**e**), and 60 (**f**) round fish fingerlings (Serrasalmidae).

A total of 390 color photographs of round fish fingerlings (Serrasalmidae) were captured in an illuminated environment using a 12-megapixel, 4608 × 2592 resolution iPhone XR smartphone camera. The files were divided into equal numbers and organized into work folders, each containing 65 images, and named according to the number of fish present in the photos (9, 20, 30, 40, 50, and 60 fingerlings) (Figure 2). A fraction of 70% of the photos was used for training, 20% for validation, and 10% for testing the CNN as proposed by Malcher and Guedes [23] and Vendruscolo [24] and then compared for performance.

### 2.2. Pre-Processing and Labeling the Fish

**Figure 3:**
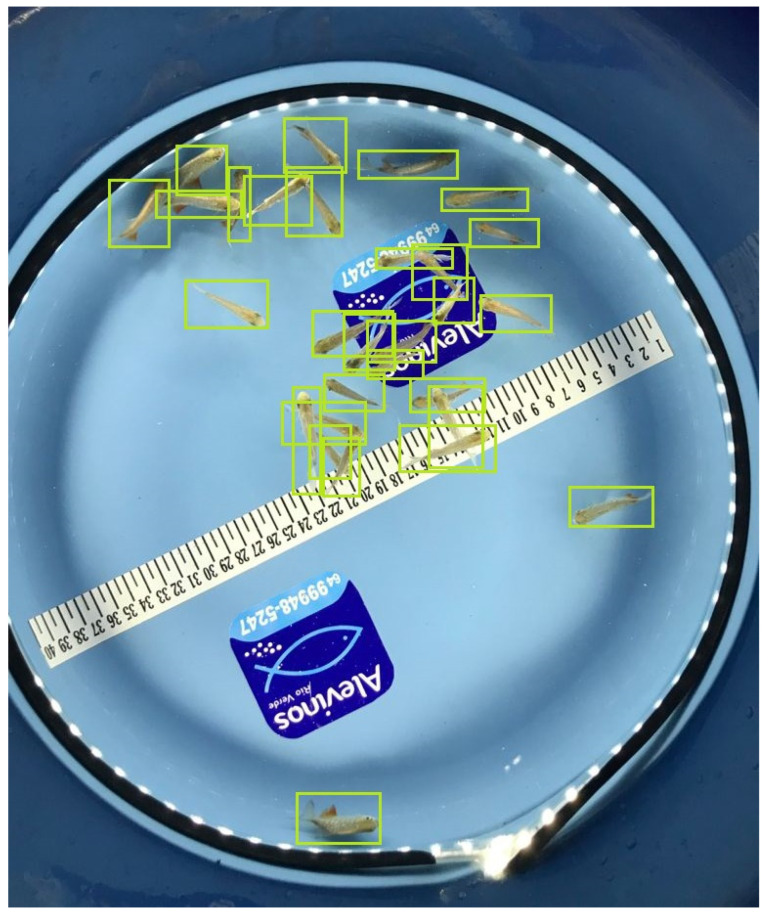
Masks around the fish fingerlings made through LabelImg.

After collecting and organizing the data, the fish were identified and marked with bounding boxes (masks) on each image using the LabelImg (https://docs.roboflow.com/annotate/use-roboflow-annotate/model-assisted-labeling, accessed on 1 February 2024) graphical tool (Figure 3). 

Data labeling was conducted in a collaborative manner, involving the use of a platform that optimizes and simplifies the process. Loading of data is followed by a crucial pre-processing phase. At this stage, techniques for resizing, normalizing, and converting formats were applied, guaranteeing uniformity and quality in the data. The labeling team carried out a visual analysis, marking the fish identified in the images. Subsequent review and correction by the supervision team ensured the consistency and accuracy of the labels. The cross-validation stage, in which team members reviewed each other’s labels, promotes agreement and accuracy. Once completed and validated, the labeled dataset was exported in the desired format, ready to be used to train machine learning models. Collaborative supervision by the team provided a robust approach, drawing on diverse perspectives. The software employed simplified the process, facilitating efficient coordination throughout the data labeling cycle and contributing to the reliability of the labels.

This step is crucial for the success of the neural network regardless of the framework used, because it is through labeling that network learning occurs [25]. Thus, the correct selection of the boxes is a determinant of effectiveness in recognizing and counting fish [16].

Data augmentation was used, which involves introducing variations to existing images or training data, providing fundamental diversification to the dataset. This, in turn, improves the model’s ability to generalize effectively to unobserved data. In short, data augmentation has emerged as a valuable strategy for strengthening the generalizability of deep learning models, especially in data-limited scenarios. It offers an effective solution to the challenges associated with overfitting and improve the robustness of the model in face of variations in real data.

### 2.3. Fish Detection and Counting

For the detection and counting of fish, two models with detection algorithms were used, and their effectiveness were compared. One of them was an open-source, free license, and real-time detection CNN proposed by Bochkovskiy et al. [26] and was freely available on GitHub (https://github.com/, accessed on 1 February 2024)/Google Colab (https://colab.research.google.com, accessed on 1 February 2024). The other one has an online platform, is consolidated in the market, and can operate in various environments to solve complex problems in an automated and didactic way [27,28]. Data processing was performed on a computer with an Intel Core i5-10400.2.90 GHZ × 12 processor (Intel, Santa Clara, CA, USA), with 32 gigabytes (GB) of RAM (Dell Inc., Round Rock, TX, USA) and 240 GB of solid-state disk (SSD) (Kingston Technology, Fountain Valley, CA, USA) storage, and the total number of iterations of all the training data was 200 epochs.

**Figure 4:**
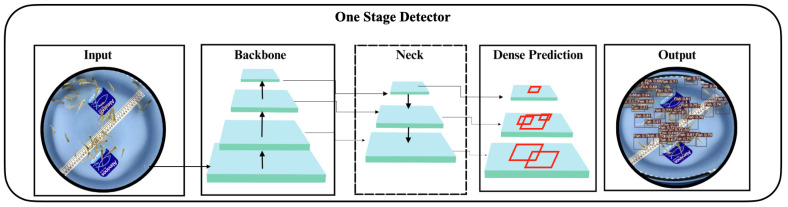
Architecture of the first convolutional neural network used. Adapted from [28].

The architecture of the first convolutional neural network used consists of 415 layers that formed a deep model for object detection. Initially, the input was processed by convolutional layers, where the first layers (indices 0 to 4) performed convolutions with different filters to extract low-level features. Layers 5 and 6 perform additional convolutions and were connected to layer 4 via a concatenation. The next block (indices 7 to 22) repeated convolutions with deeper layers, followed by a concatenation in layer 10. This pattern was repeated with some variations to build a richer representation of features. Pooling layers (indices 12, 25, 38, 51, 76, and 89) were used to reduce spatial dimensionality and preserve the most important features. Subsequent layers (indices 43 to 62) included convolutions and concatenations to form an intermediate block before connecting to higher resolution layers. Layers 63 to 75 applied additional convolutions and use up sampling to increase spatial resolution. The final part of the architecture (indices 76 to 105) involved repetitive blocks of representative convolutions (RepConv) and ended with the IDetect layer, which was specific to object detection. This IDetect layer used information about the dimensions of the grid and the sizes of the boxes to perform object detection at different scales. This architecture, illustrated in Figure 4, reflects a complex configuration, where the network learns to represent hierarchical features at various scales to perform the task of object detection. The use of convolutional layer repetitions and concatenations contributes to the network’s ability to learn rich and complex representations. Table 1 below shows the main parameters used to configure the neural network.

The model employed in the paid platform adopts an AutoML (automatic machine learning) approach, and although it does not disclose the underlying architecture, it offers a notable advantage in the versatility and simplicity in the process of training computer vision models. Its main feature is the ability to provide an accessible and user-friendly environment, which is especially designed for users without extensive experience in machine learning. This simplified approach makes it considerably easier to train customized models for specific computer vision tasks. The platform prioritizes usability and adaptability, making it an attractive choice for those who want to obtain custom models without the need for an in-depth understanding of the intricate details of machine learning.

### 2.4. Evaluation Metrics

The performance of a CNN in object recognition was estimated by evaluation metrics. Commonly, the metrics were derived from a confusion matrix that categorizes the model’s hits and misses in rows and columns into four variables: true positive (TP) when correctly identified, false positive (FP) when the object is wrongly detected, true negative (TN) when a result in which the model correctly predicted the negative class, and false negative (FN) when a result in which the model incorrectly predicted the negative class [29].

**Figure 5:**
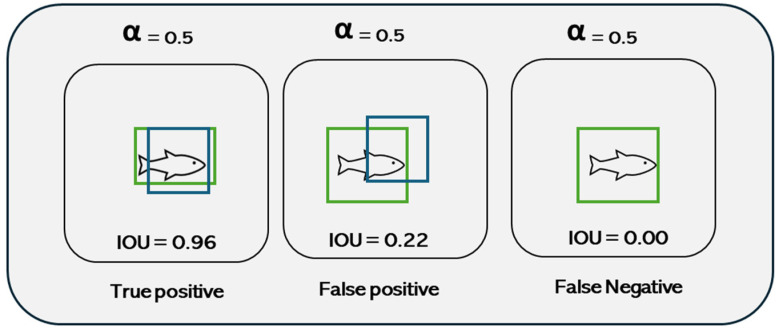
Graphical representation of the intersection of the manual bounding box (green) and the blue bounding box predicted by the neural networks. When the overlap (IoU) between them is 50%, the CNN identified the object as a hit.

The identification of the CNN model used was based on the intersection between the bounding boxes made manually around the object and the bounding boxes predicted by the neural network (Figure 5). It was considered a hit when the intersection over union (IoU) of predicted and demarcated boxes reached the threshold of 50% (mAP@0.5) [30].

From these variables, the accuracy (A), precision (P), recall (R), and F-Score were calculated. The accuracy revealed the ratio of correct predictions in the test set considering all elements identified in the images, whether true or false. Precision indicated the TP hit, i.e., the percentage of identified fish fingerling that were truly fish. Recall or sensitivity represented the number of fish in the image recognized by the model, consequently revealing which were not identified (FN). The F-Score was the weighted average of precision and recall. In this work, balanced data with a class to be identified (fish) were used, and the metrics used to evaluate the performance of the networks were:

(1) $$A=\left(TP+TN\right)/\left(TP+TN+FP+FN\right)$$

(2) $$P=TP/\left(TP+FP\right)$$

(3) $$R=TP/\left(TP+FN\right)$$

(4) $$F-Score=2*\left(P*R\right)/\left(P+R\right)$$

The approach adopted was strategically balanced, prioritizing the minimization of false negatives (FNs) and false positives (FPs) in a similar way, recognizing the equivalent importance of both types of errors. To implement this strategy, we used the threshold adjustment, modifying the model’s decision threshold. This action aims to balance the rate of FNs and FPs, ensuring that neither type of error was favored over the other. Threshold manipulation is an effective technique for personalizing the model’s decisions. It is sensitive to the specific context of the application and tolerant of different types of mistakes. This balanced approach was adopted to ensure robust performance, while also considering the implications of false negatives and false positives in the application scenario.

## 3. Results

The two evaluated models achieved a mAP above 0.8 (80%). According to the methodology, a minimum mAP of 0.5 was required. The results indicated that the neural networks were effective in identifying Serrasalmidae fingerlings using 200 epochs for training.

The accuracy, precision, recall, and F-Score were above 99% for all fish densities for the CNN, with higher values observed for lower densities. For the AutoML model, precision (P) was above 99% for all fish densities, but accuracy and recall decreased from 86% to 59% according to the fish density (10 to 60 fish, respectively). The F-Score also decreased with an increase in fish, ranging from 92.5% to 74% for AutoML (Table 2). 

**Figure 6:**
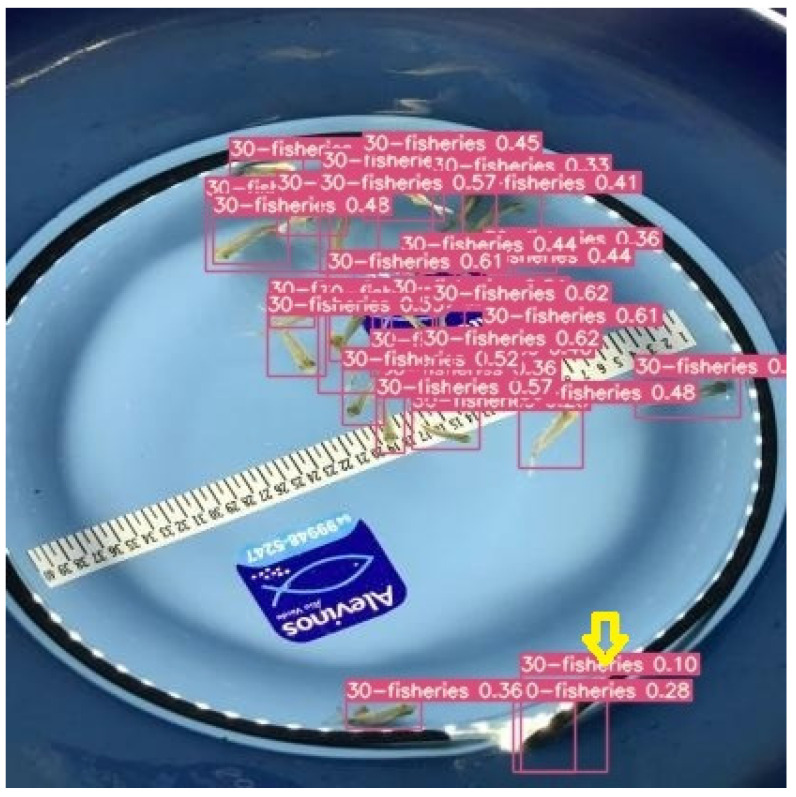
The yellow arrow indicates the false positive (FP) identified by the CNN model.

The increase in the number of animals compromised the sensitivity of the AutoML model, reflecting a considerable increase in false negatives (FNs) and causing a direct and negative impact on accuracy, recall, and F-Score. In cases where the network’s precision was 100%, such as in photos of 10, 30, 40, 50, and 60 fish submitted to AutoML, the model did not identify false positives (FP = 0), meaning that all images identified with fingerlings were indeed fingerlings. However, the low sensitivity or recall, from 86% to 43%, directly affected the accuracy of the AutoML model, resulting in a sharp drop of densities of 30 fish or more. The worst performance in images with higher fish densities may be related to animal agglomerations that led to fish overlapping in the image. On the other hand, the CNN’s high sensitivity (above 99%) and speed resulted in lower precision compared to the AutoML model, causing, for example, the container’s edge to be identified as a fish (Figure 6).

Nevertheless, the CNN model presented better performance when evaluating the metrics together, maintaining sensitivity and precision balanced with very low FNs, while controlling the incidence of FPs in all image categories, achieving rates above 99% in the four evaluated metrics.

## 4. Discussion

Costa et al. [31] achieved a mean average precision (mAP-0.5) of 97.30%, but they used 12 and 24 epochs. The use of fewer epochs reduces unnecessary expenses on computational resources. This result supports the idea that metrics for convolutional neural networks should be evaluated integrally and not separately, depending on the objective for which they were designed and trained [32,33,34].

Convoluted neural networks model used employ algorithms with high detection speeds that extract image characteristics in a single step, reducing interactions. This disfavors precision but allows for real-time object detection [35]. The trend towards faster detection speeds is desirable for monitoring and interventions to be performed dynamically in real-time through automation and the IoT in Industry 4.0 [36]. However, this model maintained a precision rate above 99.5%. It can recognize, classify, and distinguish objects in a single step with high accuracy and sensitivity. It is more efficient than other CNN’s, other computer vision algorithms, such as the RCNN and Fast RCNN, and traditional fish biometric methods [35,37,38]. Achieving an accuracy of 99% or higher was a significant result for the CNN model. 

Park and Kang [39] considered neural networks with 97% accuracy in identifying fish in underwater images as high performing, based on a dataset of 5,000 images. In another study, Cai et al. [40] evaluated different neural networks to identify fish with spring viremia of carp disease. They found that it was necessary to include an MPDIoU loss function to obtain results above 95%. In their work, they used a dataset of 1814 images (training: 1450; test: 364) out of a total dataset of 10,000 images.

Our study utilized fewer images compared to other authors, resulting in reduced efforts for image collection, labeling, computational costs for training, and testing. The success of the neural network can be attributed to its characteristics and the high image quality.

Sharpness of the object to be identified and quality of the image were crucial for training the neural network. Developing a high-precision target detection neural network model requires a good dataset as a preliminary requirement. In an aquatic environment, images may be distorted, blurred, or have color distortion due to factors such as water refraction, dispersion, and color attenuation [41]. However, in this study, none of the collected images were excluded. Data was collected under controlled experimental conditions with clean water, making the fry easily visible. Other studies conducted with fish in similar environmental conditions have also achieved accuracies of over 95% [18].

This study suggests that the first neural network used was more effective in counting fish, and it was not affected by the overlapping or aggregation of fish, as accuracy remained unchanged even when the density of fish increased. The second model was unable to achieve this, as accuracy decreased with an increase in the density of fish per image. 

The experimental conditions and the technological tool developed for counting Serrasalmidae fingerlings can be easily applied in units that sell them, particularly in retail units, as a replacement for the current method of manual counting through sampling. In these commercial units, the animals are counted and packaged for transportation. This process was conducted in a clean water environment.

## 5. Conclusions

It can be concluded that convolutional neural networks were effective in detecting and counting fish fingerlings in images. However, the results may vary depending on the specific neural network used, particularly in relation to different quantities of fish. The first model based on a CNN showed excellent performance in recognizing fish fingerlings, with accuracy, precision, and recall rates equal to or greater than 99%, regardless of fish density. However, the AutoML model’s accuracy, precision, and recall rates decreased as the number of fish increased.

## Figures and Tables

**Table 1:** Parameters used to configure the neural network.

Implementation Details	Parameters
Training	r0 = 0.01, lrf = 0.1, momentum = 0.937 weight_decay = 0.0005 box = 0.05, loss_ota = 1 Batch size = 2 Max-epochs = 200 Loss_function = BCE (Binary Cross Entropy) Input_size = 768 × 1024 IOU_thres = 0.45
Environment	CUDA:0 (Tesla T4, 15,102.0625 MB) Platform = Python 3.8 Implementation tools = PyTorch

**Table 2:** Accuracy, precision, recall, and F-Score of CNN and AutoML models in the identification of Serrasalmidae fingerlings by images at densities of 10, 20, 30, 40, 50, and 60 fish.

Fish	Accuracy		Precision (%)		Recall (%)		F-Score (%)	
Number	CNN	AUTOML	CNN	AUTOML	CNN	AUTOML	CNN	AUTOML
10	99.0	86.0	100	100	99.0	86.0	99.5	92.5
20	100	79.7	100	99.5	100	80.0	100	89.0
30	99.0	61.0	99.7	100	99.4	61.0	99.5	75.8
40	99.3	52.0	99.8	100	99.5	52.0	99.6	69.0
50	99.3	43.0	99.8	100	99.5	43.0	99.6	60.1
60	99.4	59.0	100	100	99.4	59.0	99.7	74.0

## Data Availability

Data will be made available on request.
